# A Model Clergyman

**Published:** 1851-12

**Authors:** 


*A Model Clergyman.*— At a semi-centennial celebration of the settlement of the Rev. Dr. Snell, over the church in North Brookfield, Mass., the Rev. Dr., in addressing his people, took occasion to remark: "One other thing I must not suppress; I would patronize regular-bred physicians — men of good character, and well acquainted with their profession. It is perfectly preposterous to suppose that those who never made the human system, and diseases and medicine, their study, should better know what ails the patient, and what treatment his case, under all circumstances requires, than observation and practice. Health and life are too valuable to be sacrificed on the shrine of ignorance. I would have no fellowship with ultraism, humbuggery, quackery, mesmerism, and mysterious knockings — all of a sort — the plague of wise men and the idols of fools." We commend this to the notice of our clerical friends who are so anxious to make proselytes to the modern systems of medical tom-fooleries.— *N. Y. Med. Times.*

